# The cytokine structural homologs IL-1β and IL-1Ra have distinct dynamical character that potentially influence their roles in allosteric regulation of the IL-1 receptor

**DOI:** 10.64898/2026.09.10.750673

**Published:** 2026-09-14

**Authors:** Glorisé Torres-Montalvo, Taylor R. Cole, Anthony C. Bishop, Rosana Lopes, A. Joshua Wand

## Abstract

Interleukin-1β (IL-1β) and the interleukin-1 receptor antagonist (IL-1Ra) share the β-trefoil fold, engage the same receptor (IL-1R), but produce opposite biological outcomes. IL-1β recruits the accessory protein IL-1RAcP to initiate inflammatory signaling while IL-1Ra occupies the receptor without supporting co-receptor recruitment. Seeking the origin of this divergence in properties not apparent from static structures, we used solution NMR relaxation to characterize fast internal motion in both proteins. Rigorous treatment of macromolecular tumbling shows both to be nearly maximally triaxial, which admits two near-degenerate axially symmetric solutions. This ambiguity was propagated throughout and found to have negligible impact. Complete replicate data sets from independently prepared IL-1β samples establish the experimental precision directly. Backbone amide and methyl-bearing side chain order parameters are indistinguishable between the two proteins, the latter averaging 0.484 ± 0.030 and 0.486 ± 0.033, indicating essentially equivalent residual conformational entropy. Their spatial distributions are uncorrelated when projected onto the shared fold. Importantly, the difference between the proteins is concentrated at the receptor interface, where methyl probes lying on the IL-1β footprint are considerably more mobile than those of IL-1Ra. It is therefore the agonist, not the antagonist, that presents an anomalously mobile binding surface. IL-1Ra also buries some 16% less surface while ostensibly binding IL-1R more tightly, the signature of an interface predisposed toward productive contact. Conformational entropy can thus distinguish protein function where structure and average flexibility are conserved, and the distinction resides not in how much entropy a protein has but in where it is kept.

## Full Text Availability

The license terms selected by the author(s) for this preprint version do not permit archiving in PMC. The full text is available from the preprint server.

